# Hyperspectral Imaging-Based Evaluation of Seasonal Growth Characteristics in Turfgrass

**DOI:** 10.3390/plants15091393

**Published:** 2026-05-01

**Authors:** Jae Gyeong Jung, Eun Seol Jeong, Jae Yeob Jeong, Jun Hyuck Yoon, Donghwan Shim, Eun Ji Bae

**Affiliations:** 1 Forest Biomaterials Research Center, National Institute of Forest Science, Jinju 52817, Republic of Korea; rtyhj6@gmail.com (J.G.J.); jeojy003@korea.kr (J.Y.J.);; 2 Department of Biological Sciences, Chungnam National University, Daejeon 34134, Republic of Korea; dshim104@cnu.ac.kr

**Keywords:** hyperspectral imaging, high-throughput phenotyping, *Zoysia*, vegetation indices, breeding population, turfgrass, image segmentation

## Abstract

Efficient phenotyping is essential for accelerating genetic improvement in turfgrass breeding, where manual measurements are labor-intensive. This study evaluated hyperspectral imaging (HSI) as a high-throughput tool for assessing *Zoysia* spp. breeding populations consisting of 464 genotypes. HSI data (400–1000 nm) were processed through a user-in-the-loop hybrid segmentation pipeline integrating UMAP dimensionality reduction, DBSCAN clustering, Random Forest classification, and pseudo-RGB refinement. To independently assess vegetation classification performance, 10,000 manually annotated reference points from 50 pseudo-RGB images were compared with the automated module, yielding an overall accuracy of 0.9697, a precision of 0.8830, a recall of 0.9240, a specificity of 0.9779, an F1-score of 0.9030, and Cohen’s kappa of 0.8851. A Combined Ranking Score (CRS) integrating five vegetation indices and vegetation pixel count was significantly associated with aerial shoot count (*r* = −0.445, *p* < 0.001) and runner count (*r* = −0.207, *p* < 0.001). The highest-ranked genotype showed a 9370.3-pixel increase in vegetation area between 6 and 16 weeks after transplanting, compared with 1417.7 pixels for the lowest-ranked genotype. Classification performance declined under high-coverage conditions, indicating increased mixed-pixel ambiguity in dense canopies. These results suggest that HSI-based CRS can support rapid, objective, and non-destructive relative ranking of density-related vegetative growth in turfgrass breeding. Because the study was conducted at a single location and season and correlations with manual traits were moderate, the framework is best interpreted as a screening and ranking tool rather than a direct predictive model.

## 1. Introduction

*Zoysia* spp. (Poaceae) are perennial warm-season turfgrasses distributed primarily in East Asia, Australia, and the Pacific Rim, with approximately 11 species reported worldwide. In Korea, five species—*Zoysia sinica*, *Z. japonica*, *Z. macrostachya*, *Z. matrella*, and *Z. pacifica*—are distributed natively across diverse habitats, including coastal areas, inland grasslands, and mountainous regions [1]. Zoysia grasses are valued for their superior tolerance to wear, salinity, and drought compared to cool-season grasses, making them low-maintenance options. However, a physiological trade-off exists between stress tolerance and growth rate, resulting in relatively slow recovery [2,3]. Most Zoysia cultivars are established vegetatively through sod, or runners, as seed production is extremely limited or commercially unavailable [4]. In commercial sod production, fields can take several months to reach harvestable maturity, making the selection of high-performing vegetative lines critical for both production efficiency and market competitiveness. Therefore, efficient identification of superior genotypes with enhanced vegetative growth characteristics is essential not only for breeding programs but also for practical sod industry applications.

Image analysis technology in turfgrass research has focused on non-destructive growth diagnosis, starting with digital image analysis (DIA) proposed by Richardson et al. [5]. Image-based phenotyping offers several advantages over traditional destructive methods or visual ratings. First, it allows for non-destructive measurement, enabling repeated measurements of the same individual, which is essential for studying growth dynamics. Second, it facilitates the collection of objective and quantitative data, minimizing rater bias. Third, it enables the rapid processing of large sample sizes, making it suitable for high-throughput phenotyping (HTP) required in breeding programs. Recently, unmanned aerial vehicles (UAVs) have been applied to large-scale turfgrass research. Zhang et al. [6] reported a high correlation (*R*^2^ = 0.90) between UAV-based NDVI and ground-measured NDVI in Bermuda and *Zoysia* variety trials, confirming an 80–92% agreement in selecting top-performing cultivars. Hernandez et al. [7] developed a turfgrass quality prediction model using multispectral and thermal images combined with machine learning (Ordinal Forest), demonstrating that objective models (multi-class AUC = 0.872) can complement the subjectivity of visual evaluations. Objective imaging-based screening has also been demonstrated in zoysiagrass, where high-resolution RGB and multispectral imagery were used to estimate green cover and identify cold-tolerant materials [8]. In addition, machine learning-based imaging systems have recently enabled efficient evaluation of drought-response phenotypes in a bentgrass hybrid population of approximately 1000 lines [9].

Hyperspectral imaging (HSI) provides richer spectral information than multispectral imaging by utilizing hundreds of continuous narrow bands [10,11,12]. This high spectral resolution allows for the precise estimation of physiological and biochemical traits, such as chlorophyll content, water status, and nitrogen concentration. In a comparative analysis of drought stress in Kentucky bluegrass, HSI-based vegetation indices showed the highest coefficients of determination for predicting turfgrass quality (TQ) and relative water content (RWC) (*R*^2^ = 0.90 and 0.88, respectively) [13]. This study compared hyperspectral, multispectral, and chlorophyll fluorescence imaging approaches. Notably, indices such as SIPI and SRI were identified as the most sensitive for early drought detection. Previous studies have also presented the feasibility of quantifying turfgrass quality through chlorophyll estimation models (*R*^2^ = 0.817) using hyperspectral reflectance [14] and confirmed correlations with drought stress [15], suggesting that HSI technology is highly applicable to precision turfgrass management. More recently, hyperspectral phenomics has also been applied to zoysiagrass breeding materials using sUAS-based imaging, indicating that breeding-oriented hyperspectral workflows in turfgrass are now emerging [16].

However, most existing studies have been conducted on a small scale or limited to a few cultivars under specific stress conditions; cases applying HSI-based HTP to large-scale genetic resources are extremely rare. In particular, evaluating the growth characteristics of F1 segregation populations, which can number in the hundreds, using conventional visual assessment or destructive analysis is realistically constrained. On the other hand, while UAV-based hyperspectral systems are advantageous for wide-area coverage, they are often limited by the complexity of radiometric calibration due to varying weather and illumination conditions [17]. Moreover, trade-offs between flight altitude, spatial resolution, and sensor payload capacity restrict their applicability for the precise, centimeter-scale analysis required in breeding plots [18]. In contrast, portable (proximal) hyperspectral devices can acquire high-resolution spectral information through close-range imaging, making them suitable for evaluating breeding populations where planting intervals are narrow or precise analysis of small plot units is required. This gap is particularly important in turfgrass breeding, where high-throughput phenotyping must operate at the genotype scale while still capturing physiologically meaningful spectral traits under field or nursery conditions [9,16].

Since hyperspectral images consist of high-dimensional data with hundreds of bands, simple thresholding or index-based masking can be unreliable when soil and shadow spectra overlap with those of vegetation [19,20]. In particular, the high dimensionality of hyperspectral data can reduce the stability and efficiency of conventional classifiers operating directly on raw spectral bands [21,22]. Therefore, a preprocessing step that visualizes data structure and reduces redundancy using non-linear dimensionality reduction techniques, such as Uniform Manifold Approximation and Projection (UMAP), is useful for practical ROI extraction from field-acquired hyperspectral data [23]. Recently, hybrid approaches combining unsupervised learning (Clustering) and supervised learning (Classification) have been attempted to improve the accuracy of Region of Interest (ROI) extraction.

In conventional plant phenotyping, pixel-wise classification has been widely used to precisely separate leaves from the background. For instance, studies have employed Support Vector Machine (SVM) or Random Forest (RF) algorithms to identify stress types or selectively extract stress-affected areas [24,25], or applied deep learning-based semantic segmentation [26,27]. However, for HTP of large breeding populations, fully supervised deep learning workflows often require large pixel-level annotated datasets and may not generalize well across crops and field conditions [28,29,30]. In this context, a user-in-the-loop hybrid pipeline based on unsupervised clustering and conventional classification can offer a practical alternative by enabling flexible correction while maintaining a relatively low annotation burden [27,28,31]. Building upon such pixel-level segmentation, it becomes possible to extract quantitative vegetation information that can be directly compared with conventional manual measurements.

Conventional growth evaluation of turfgrass relies on manual measurements such as plant height, aerial shoot counts, and runner enumeration. However, these methods are labor-intensive, time-consuming, and subject to inter-rater variability, making them impractical for large-scale F1 population screening. If spectral vegetation indices can be shown to correlate strongly with these growth parameters, HSI-based assessment could serve as a rapid, objective, and non-destructive alternative for HTP in breeding programs.

Therefore, this study aimed to evaluate whether portable VNIR hyperspectral imaging can support high-throughput phenotyping of seasonal growth characteristics in a large Zoysia breeding population. Specifically, this study contributes in three ways: first, by applying proximal HSI to a large turfgrass breeding population under field nursery conditions; second, by proposing a Combined Ranking Score (CRS) that integrates vegetation quality and quantity; and third, by implementing a practical user-in-the-loop hybrid segmentation workflow for breeding-oriented image analysis. We further examined whether HSI-derived ranking results were associated with conventional growth traits, thereby assessing the utility of this framework as a rapid and non-destructive relative selection tool.

## 2. Results

### 2.1. Image Segmentation Results

**Figure 1:**
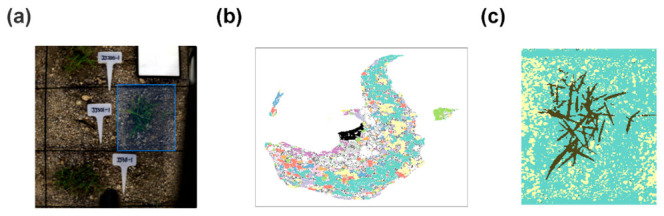
Image processing workflow for precise turfgrass segmentation: (**a**) ROI selection from the hyperspectral image. (**b**) Visualization of spectral feature clustering using UMAP and DBSCAN. Distinct colors represent different spectral clusters, while the black points indicate the selected turfgrass cluster. (**c**) Final segmentation output generated by Random Forest classification. The background (excluding the black turfgrass region) indicates non-target regions (soil, shadow) masked out from the analysis.

Accurate vegetation segmentation is critical for determining data quality in hyperspectral image-based field phenotyping. In this study, a hybrid segmentation pipeline combining unsupervised learning with high-dimensional spectral information and color filtering was applied in a stepwise manner (Figure 1).

First, regions of interest (ROIs) were defined for each individual plant within the image (Figure 1a), and UMAP was applied to visualize the high-dimensional spectral data. DBSCAN clustering was then performed to group pixels with similar spectral characteristics (Figure 1b). Based on this grouping information, a trained Random Forest Classifier (RFC) identified turfgrass regions in the primary classification step (Figure 1c). During this process, vegetation areas were overlaid in black, confirming that vegetation and non-vegetation pixels were generally separated into distinct spectral clusters according to their spectral properties.

**Figure 2:**
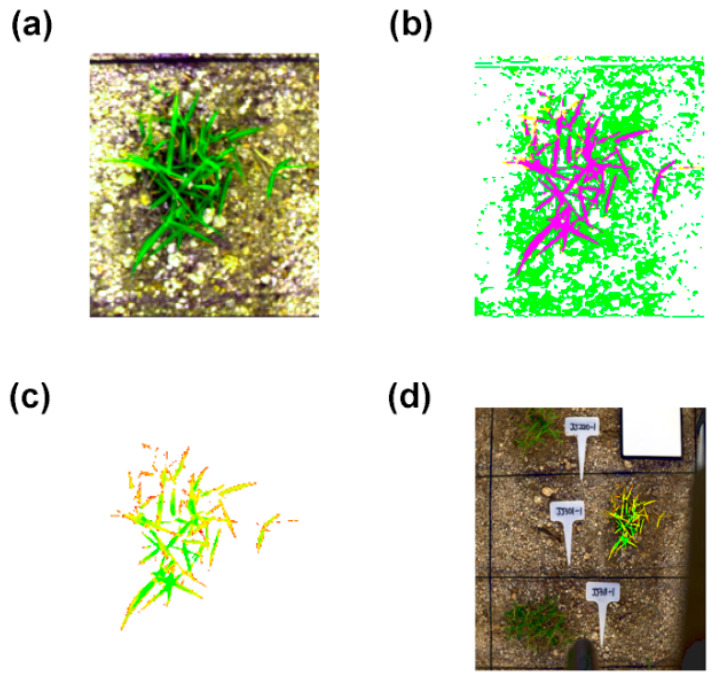
Refinement of segmentation via RGB intersection masking and vegetation index mapping: (**a**) Original pseudo-RGB image of the target turfgrass. (**b**) Visualization of the intersection masking process. White represents regions detected by neither RF nor RGB; green pixels indicate areas detected only by RGB thresholding (noise); yellow pixels indicate areas detected only by RF classification; and magenta pixels represent the valid intersection (RFC ∩ RGB) retained for analysis. (**c**) Extracted vegetation pixels after applying the intersection mask. (**d**) Final spatial visualization of vegetation indices overlaid on the original image using the refined mask coordinates.

To further improve classification precision, a refinement process using the intersection of RFC results and pseudo-RGB color information was performed (Figure 2). Target RGB values corresponding to actual turfgrass as perceived by the user were extracted from the pseudo-RGB image (Figure 2a), and only the magenta regions where pixels within the tolerance range matched the RFC results were confirmed as the final mask (Figure 2b). This intersection masking technique effectively removed fine background noise that the RFC model might miss, enabling more accurate delineation of the morphological structure of vegetation.

Finally, spectral reflectance was extracted only from regions corresponding to the morphological structure of vegetation, and vegetation indices were calculated and visualized (Figure 2c). These results were then overlaid onto the original captured image (Figure 2d). This analysis enabled clear visualization of the physiological status of each individual with substantially reduced interference from background noise or soil. Furthermore, even when multiple genotypes were captured in a single image, the ROI-based approach enabled the separate extraction of vegetation data for each individual, suggesting that this pipeline can efficiently provide phenotypic data for large-scale field evaluations.

To provide an independent assessment of the vegetation classification module, 50 pseudo-RGB images from the representative validation set were used, and 200 random pixels were sampled from each image, yielding 10,000 manually annotated reference points in total. Overall, the automated classification achieved an accuracy of 0.9697, a precision of 0.8830, a recall of 0.9240, a specificity of 0.9779, an F1-score of 0.9030, and Cohen’s kappa of 0.8851 (Table 1). Performance varied with canopy coverage, with lower F1-scores in high-coverage images than in intermediate- and low-coverage images, indicating that dense canopy conditions remain more challenging for accurate vegetation/background discrimination.

### 2.2. Spectral Reflectance of Selected Groups

**Figure 3:**
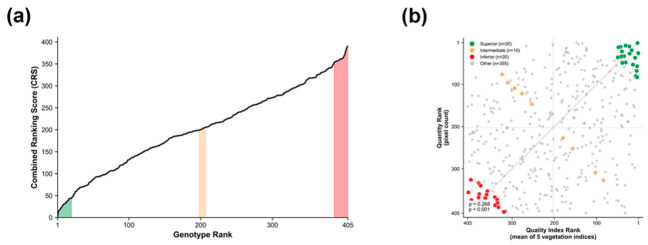
Selection of representative genotypes based on the Combined Ranking Score (CRS): (**a**) Distribution of CRS across 405 genotypes. Shaded regions indicate selected representative groups: Superior (green, Rank 1–20), Intermediate (orange, Rank 198–207), and Inferior (red, Rank 386–405). (**b**) Relationship between quality index rank (mean of five vegetation indices) and quantity rank (pixel count). The dotted line represents the 1:1 relationship. Spearman’s correlation (ρ = 0.268, *p* < 0.001) indicates a low but statistically significant positive correlation, supporting the integration of both metrics into CRS.

The Combined Ranking Score (CRS), integrating vegetation indices and growth area, was calculated for 405 genotypes, and the population exhibited a wide range of phenotypic variation. Prior to rank integration, individual vegetation indices showed substantial rank variation for the same genotype, with an average rank range of 55.4 positions and a maximum of 213 positions across the five indices. Such variability demonstrates that selection based on any single index would be highly sensitive to index choice; therefore, rank aggregation across multiple indices was employed to provide a consensus ranking robust to arbitrary index selection. Based on this ranking, 50 representative genotypes were selected to capture the range of diversity. The selected population was stratified into three groups: Superior (Top 20, within the top 5% of CRS), Intermediate (Middle 10), and Inferior (Bottom 20, within the bottom 5%) (Figure 3a). This stratified sampling approach represents the diversity of the germplasm collection and provides baseline data for spectral validation.

The relationship between quality index rank (mean of five vegetation indices) and quantity rank (pixel count) showed a low but statistically significant positive correlation (ρ = 0.268, *p* < 0.001), indicating that these two metrics capture largely independent aspects of plant performance (Figure 3b). Genotypes with high vegetation index values did not necessarily exhibit large canopy coverage, and vice versa. The observed independence justified the integration of both metrics with equal weighting (50:50), as relying on either metric alone would overlook complementary information.

**Figure 4:**
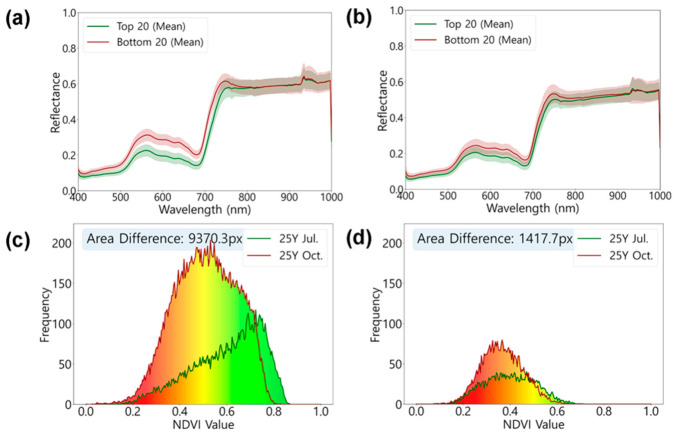
Comparison of spectral characteristics and vegetation abundance between growth stages: (**a**,**b**) Mean spectral reflectance of the Top 20 and Bottom 20 groups in July (**a**) and October (**b**). Solid lines represent the group average, and shaded areas indicate ± 1 standard deviation (SD). (**c**,**d**) Histograms of NDVI frequency distribution for representative individual genotypes: Rank 1 (**c**) and Rank 405 (**d**).

To characterize the physiological differences between the Top 20 and Bottom 20 groups, mean spectral reflectance in the visible and near-infrared (VNIR) region was analyzed (Figure 4a,b). The two groups showed different reflectance patterns in the visible wavelength range (400–700 nm). The Top 20 group exhibited lower reflectance than the Bottom 20 group across the visible region, with this difference being more apparent in the green and red wavelengths. In October, the Top 20 group maintained similar reflectance levels, whereas the Bottom 20 group showed decreased reflectance, resulting in a reduced gap between the two groups.

To quantify temporal changes in growth characteristics according to performance rank, NDVI frequency distributions were compared between July and October (Figure 4c,d). The top-ranked individual (Rank 1) exhibited high NDVI values ranging from 0.6 to 0.8 in July, with the distribution shifting leftward by October. Despite this shift, the vegetation area increased by 9370.3 pixels, suggesting canopy expansion during the growing season. In contrast, the lowest-ranked line (Rank 405) showed low NDVI values (0.3–0.5) and limited vegetation area from the initial growth stage in July. By October, this genotype exhibited an increase of only 1417.7 pixels, indicating lower growth compared to the top-ranked individual.

### 2.3. Vegetation Indices Comparison

**Figure 5:**
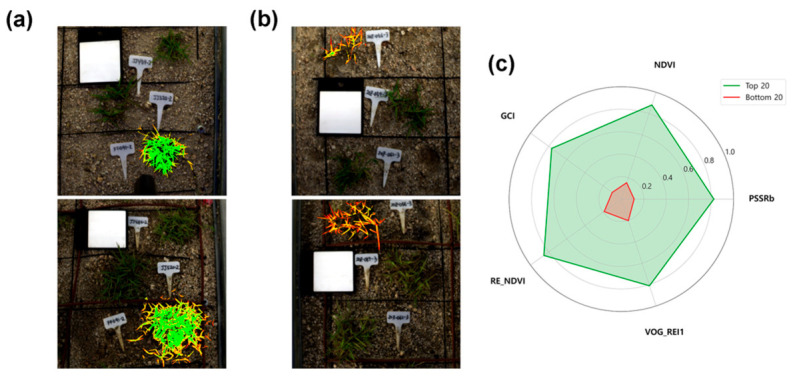
Visual comparison and vegetation index profiles of selected genotypes: (**a**,**b**) Representative RGB images with vegetation index overlay for the highest-ranked (Rank 1, (**a**)) and lowest-ranked (Rank 405, (**b**)) genotypes in July (top) and October (bottom). (**c**) Radar chart comparing the mean values of five vegetation indices between the Top 20 and Bottom 20 groups.

To verify whether the CRS-based ranking reflects actual growth status, visual comparison and vegetation index analysis were performed for the highest-ranked (Rank 1) and lowest-ranked (Rank 405) individuals (Figure 5).

Vegetation indices were overlaid on RGB images to facilitate visual assessment (Figure 5a,b). The top-ranked individual (Rank 1) maintained high coverage and vegetation index values in both July and October, whereas the lowest-ranked individual (Rank 405) showed limited growth from July, and the vegetation index remained low through October despite an increase in leaf area.

The mean values of five vegetation indices (NDVI, GCI, PSSRb, VOG-REI1, and RE-NDVI) were compared between the two groups using a radar chart (Figure 5c). All indices were normalized to a 0–1.0 scale for relative comparison. The Top 20 group (green polygon) showed values ranging from 0.6 to 0.8 across all five indices, forming an expanded shape toward the outer edge of the chart. In contrast, the Bottom 20 group (red polygon) exhibited values between 0.2 and 0.3, forming a contracted shape near the center. These results indicate that the Top 20 group showed higher values than the Bottom 20 group across all vegetation indices examined.

### 2.4. Correlation with Morphological Traits

**Figure 6:**
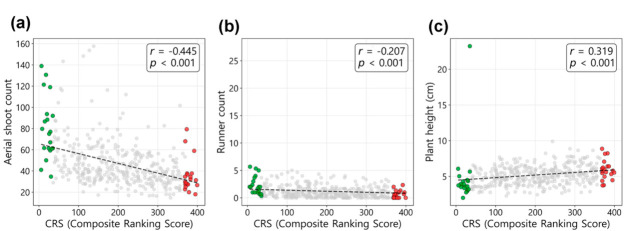
Relationship between CRS and morphological traits. Scatter plots showing the correlation between CRS and (**a**) aerial shoot count, (**b**) runner count, and (**c**) plant height across all 405 genotypes. Green and red points indicate the Top 20 and Bottom 20 groups, respectively. The dashed line in each panel represents the linear regression fit.

To examine whether the hyperspectral image-based CRS reflects morphological growth characteristics, correlations with manually measured traits (aerial shoot count, runner count, plant height) were analyzed (Figure 6). Aerial shoot count showed a moderate negative correlation with CRS (*r* = −0.445, *p* < 0.001) (Figure 6a). Lower CRS values (higher rank) were associated with a higher aerial shoot count. On the scatter plot, the Top 20 group (green points) was distributed in the upper left region (high count, high rank), while the Bottom 20 group (red points) was concentrated in the lower right region (low count, low rank). Runner count also showed a weak negative correlation with CRS (*r* = −0.207, *p* < 0.001) (Figure 6b). Although the correlation coefficient was lower than that of aerial shoot count, the Top 20 group tended to produce more runners than the Bottom 20 group. In contrast, plant height showed a weak positive correlation with CRS (*r* = 0.319, *p* < 0.001) (Figure 6c). This indicates that genotypes with lower CRS values (higher rank) tended to have shorter plant height compared to lower-ranked genotypes.

The mean morphological characteristics of the Top 20, Bottom 20, and total population are summarized in Table 2. The Top 20 group exhibited significantly higher aerial shoot count (84.1 vs. 25.9, *p* < 0.001) and runner count (2.4 vs. 0.7, *p* < 0.001) compared to the Bottom 20 group, while plant height showed no significant difference between groups (Table 2). These results support that CRS reflects density-related morphological traits rather than plant stature.

## 3. Discussion

### 3.1. Effectiveness of the Hybrid Segmentation Pipeline for Field Phenotyping

The UMAP-DBSCAN-RFC-based hybrid segmentation pipeline applied in this study demonstrated effective separation of turfgrass vegetation under the present field-nursery imaging conditions (Figure 1). Conventional thresholding methods using RGB or multispectral images have limited accuracy in environments where soil–vegetation boundaries are ambiguous or lighting conditions vary considerably [29,32,33]. Unlike RGB images, hyperspectral data are high-dimensional, making feature extraction essential to mitigate the curse of dimensionality and reduce computational complexity [22]. To address these limitations, this study utilized hyperspectral images with 204 spectral bands and employed UMAP and DBSCAN to separate background noise from vegetation pixels based on the high-dimensional spectral information (Figure 1b,c).

Notably, a ‘user-in-the-loop’ approach was implemented through intersection masking based on pseudo-RGB images (Figure 2a,b). This approach contributed to correcting misclassifications by the machine learning model. Despite recent advances in deep learning, robust and universally applicable models that can handle diverse crops and environmental conditions remain limited [30,34]. Furthermore, deep learning model development requires large-scale, accurately labeled datasets [31], and model generalizability decreases substantially when encountering new environments or weather conditions beyond the training data distribution [35]. Recent studies have demonstrated that deep learning-based segmentation can outperform conventional classifiers under variable illumination [27,28,29,30,34]; however, in the present study, we prioritized a practical user-in-the-loop workflow that allowed for flexible correction of false detections while maintaining a low annotation burden. The proposed pipeline, by comparison, provides flexibility by allowing researchers to specify target color ranges to correct model false detections. The final extracted vegetation pixels (Figure 2c) were overlaid on the original images to visually confirm the vegetation index distribution for each individual (Figure 2d). This approach may be useful for ensuring data reliability in breeding programs that require analysis of F1 populations with diverse phenotypes.

Nevertheless, the independent point-based validation revealed that classification accuracy was lower in high-coverage images, where dense canopies with overlapping leaves and internal shadows increased spectral ambiguity. Thus, the present approach should be interpreted as a pragmatic breeding-oriented solution rather than a fully generalized segmentation model.

### 3.2. Integration of Quality and Quantity Metrics in the Combined Ranking Score

When evaluating turfgrass quality, relying solely on either biomass (quantity) or physiological vigor (quality) can lead to biased selection. Current turfgrass evaluation primarily employs the visual assessment methods of the National Turfgrass Evaluation Program (NTEP) [36]; however, these methods are subjective, difficult to quantify, and largely dependent on a single metric of turfgrass quality. Instead of complex statistical models, this study proposed a simple rank-sum-based Combined Ranking Score (CRS) that is readily applicable in field conditions. CRS is calculated by summing the Quality Rank (integrated ranking of five vegetation indices) and Quantity Rank (vegetation pixel count ranking) with equal weights (50:50). The correlation coefficient between the two metrics was low (ρ = 0.268; Figure 3b), indicating that they reflect different aspects of growth characteristics. For instance, some genotypes may have a large coverage area but low physiological vigor, while others may be small but dense and healthy. Therefore, accurately selecting superior lines using a single metric alone is challenging, and CRS was designed to select genotypes that are both quantitatively abundant and qualitatively healthy by combining both metrics with equal weights. Ranking the 405 genotypes based on CRS revealed a continuous distribution across the population (Figure 3a). From this distribution, the Top 20 (Superior), Middle 10 (Intermediate), and Bottom 20 (Inferior) genotypes were selected for spectroscopic characteristic validation. In comparisons among the selected groups, the Top 20 group showed high normalized values ranging from 0.6 to 0.8 across all five vegetation indices (NDVI, GCI, PSSRb, VOG-REI1, and RE-NDVI), whereas the Bottom 20 group remained at 0.2–0.3 levels (Figure 5c).

Furthermore, the scatter plot of quality rank versus quantity rank (Figure 3b) revealed distinct patterns. While 37 genotypes ranked within the top 100 for both metrics, others showed divergent performance—excelling in either physiological quality or growth area, but not both. This pattern reinforces the rationale for the combined ranking approach, as genotypes selected solely by vegetation indices may lack adequate biomass for field establishment, whereas those selected by area alone may have lower physiological vigor and stress tolerance potential.

### 3.3. Changes in Vegetation Characteristics Between Growth Stages

Comparison between July (6 weeks after transplanting; WAT) and October (16 WAT) revealed differences in growth patterns between the two groups (Figure 4). The Top 20 group exhibited lower reflectance in the visible region (400–700 nm) compared to the Bottom 20 group, consistent with the characteristics of healthy vegetation with high chlorophyll content [37,38]. In July, the reflectance difference between the two groups was pronounced; however, by October, the visible reflectance of the Bottom 20 group decreased, narrowing the gap between groups (Figure 4a,b). This is interpreted as the lower-ranked group also growing during the growth period, with leaf color darkening and consequently reducing visible reflectance.

NDVI frequency distribution analysis showed that the top-ranked individual (Rank 1) displayed a high NDVI distribution of 0.6–0.8 in July, and although the distribution shifted leftward by October, the vegetation area increased by 9370.3 pixels (Figure 4c). This indicates that while NDVI values somewhat decreased due to seasonal changes, canopy expansion was actively occurring. In contrast, the lowest-ranked individual (Rank 405) showed a low NDVI distribution of 0.3–0.5 from July and increased by only 1417.7 pixels by October, exhibiting lower growth compared to the top-ranked individual (Figure 4d).

These differences were also confirmed in the actual images (Figure 5a,b). The Rank 1 individual maintained high coverage and vegetation indices in both July and October, whereas the Rank 405 individual had low coverage from July and showed limited growth even by October. These results suggest that CRS-based selection can capture differences in early growth status and subsequent growth trajectories, although additional validation across a larger subset and multiple environments is needed.

### 3.4. Correlation Between CRS and Morphological Traits

Correlation analysis between CRS and morphological traits indicated that the spectroscopic selection model in this study is associated with high-density traits, which are important in turfgrass breeding (Figure 6). The moderate negative correlation observed for aerial shoot count (*r* = −0.445) indicates that better-ranked genotypes (lower CRS values) exhibited higher shoot density. Similarly, runner count showed a weak but significant negative correlation (*r* = −0.207), while plant height showed no significant difference between the two groups (*p* > 0.05; Table 2), suggesting that CRS-based selection is more strongly associated with increased density (compact trait) rather than reduced plant height (dwarf trait). Since high tiller density in turfgrass is directly related to weed suppression, uniform coverage formation, and traffic tolerance [39,40], CRS may serve as a supplementary indicator for selecting genotypes with favorable density-related growth characteristics. Future studies should develop composite selection indices that additionally incorporate plant height-related traits.

### 3.5. Practical Implications for High-Throughput Phenotyping in Breeding Programs

The results of this study suggest the potential to improve the phenotyping stages of traditional breeding programs. Conventional manual surveys (such as measuring aerial shoot count and runner count) are labor-intensive and have limitations in evaluating large-scale populations of hundreds of genotypes within a short period [41,42].

Hyperspectral imaging-based HTP systems can non-destructively screen over 400 genotypes, reducing time and labor requirements. Additionally, since destructive sampling is unnecessary, repeated monitoring of the same genotypes becomes possible, which is advantageous for evaluating dynamic growth characteristics such as seasonal changes or stress responses. This approach has the potential to accelerate decision-making for the early selection of promising lines and may contribute to more efficient breeding cycles [43].

### 3.6. Limitations and Future Directions

This study has several limitations. First, the experiment was based on data from a single location and a single growing season (2025), requiring validation of reproducibility across inter-annual weather variations and diverse soil environments. Second, as the study was limited to an F1 population centered on *Zoysia japonica*, validation of applicability to other species with different leaf textures or colors, such as *Z. matrella* or *Z. pacifica*, is necessary. Third, the correlation coefficients between CRS and morphological traits (*r* = −0.207 to −0.445) have limited explanatory power, indicating that CRS has limitations as a direct prediction model for morphological traits. The CRS proposed in this study is more appropriately utilized as a relative ranking-based selection tool rather than a predictive model. Fourth, equal weighting (50:50) was chosen as a baseline to prevent bias toward either biomass accumulation or physiological vigor, as both attributes are equally critical for successful turfgrass establishment and long-term performance. Nevertheless, the weighting ratio may require adjustment depending on specific breeding objectives (growth vs. quality). Fifth, portable hyperspectral cameras have throughput limitations compared to UAV-based systems, necessitating automated image acquisition methods for application to large-scale populations of thousands of genotypes. Sixth, the independent point-based validation indicated that classification performance declined in high-coverage images compared with low-coverage images. This pattern likely reflects mixed-pixel effects caused by the limited spatial resolution of the hyperspectral images (512 × 512 pixels), particularly when vegetation and background are tightly intermixed in dense canopies. Because the present experiment was conducted in a breeding nursery with spatially separated individual plants, further validation will be necessary before extending the current workflow to dense mature turf canopies or more heterogeneous field backgrounds.

Future studies should evaluate the environmental stability of the CRS model through multi-location validation trials and compare the current user-in-the-loop workflow with more annotation-intensive deep learning alternatives [21,27,28,29,30]. Technically, based on the current ‘user-in-the-loop’ approach, it would be possible to develop pipelines that automatically optimize ROI settings and masking thresholds using accumulated data. Such automated ROI optimization strategies would further improve scalability and establish an HTP platform that can be readily utilized by breeders.

## 4. Materials and Methods

### 4.1. Plant Materials and Experimental Setup

In this study, a large-scale population comprising 464 *Zoysia* genotypes—consisting of 439 F1 breeding lines and 25 commercial or state varieties—was utilized. The experiment was conducted in cultivation benches filled with decomposed granite soil (Masato). Seedlings were transplanted between 23 May and 27 May in the form of soil-block cubes (50 mm × 50 mm × 40 mm). A drip irrigation system was installed along the perimeter of the experimental plots, providing supplemental water 1–2 times per week (30 min per session) during dry periods to prevent substrate desiccation. The plants were cultivated for a total of 16 weeks. Following transplantation, the plants were maintained outdoors for approximately 6 weeks to allow for acclimatization and recovery from transplant shock prior to the first evaluation.

### 4.2. Evaluation of Growth Characteristics

Data collection was carried out at two distinct growth stages: (1) 6 weeks after transplanting (6 WAT), serving as the baseline after stabilization, and (2) 16 weeks after transplanting (16 WAT). Growth characteristics were assessed concurrently with hyperspectral image acquisition to establish ground-truth data for validating the correlation between vegetation indices and conventional growth parameters. The primary indicators included plant height, aerial shoot count, and runner count. Plant height was determined by measuring the length of the longest leaf for each plant. Aerial shoot count was determined by counting shoots exceeding 1 cm in height above the ground, while runner count was assessed by counting the number of extending stolons.

### 4.3. Hyperspectral Image Acquisition

**Figure 7:**
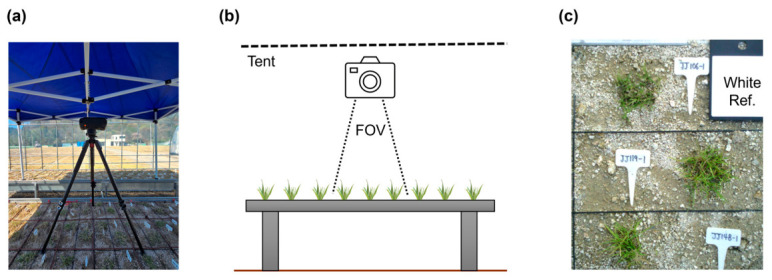
Experimental setup for hyperspectral image acquisition: (**a**) Photograph of the Specim IQ hyperspectral camera mounted on a tripod inside a portable dark tent deployed in the field. (**b**) Schematic diagram illustrating the camera position, field of view (FOV), and sample arrangement on the bench. (**c**) Representative top-view image capturing turfgrass genotypes with corresponding identification tags (ID Tag) and a white reference panel (White Ref.) used for radiometric calibration.

Hyperspectral images were acquired using a Specim IQ camera (Specim Ltd., Oulu, Finland), capable of capturing data in the visible and near-infrared (VNIR) region (400–1000 nm). The camera features a spectral resolution of approximately 7 nm and captures a total of 204 spectral bands. The sensor acquires images at a fixed spatial resolution of 512 × 512 pixels. Imaging was conducted outdoors on clear, cloudless days. The camera was mounted on a tripod at a height of approximately 1.5 m above the ground for vertical imaging (Figure 7a). To minimize shadows and diffuse reflections caused by direct sunlight, all imaging sessions were performed inside a tent. For radiometric calibration, a standard white reference panel (BaSO_4_, ~99% reflectance) supplied with the Specim IQ system was placed adjacent to the samples during every capture (Figure 7b).

### 4.4. Image Processing and Classification Pipeline

The overall hyperspectral data processing pipeline was adapted from our previous study [44], which established a robust pixel-wise classification framework integrating UMAP-based dimensionality reduction, DBSCAN clustering, and Random Forest classification. All image processing, dimensionality reduction, clustering, classification, and visualization were implemented in Python 3.13.6 (Python Software Foundation, Wilmington, DE, USA), using NumPy 1.26.4, scikit-learn 1.7.2 (for MinMaxScaler, DBSCAN, and Random Forest Classifier), umap-learn 0.5.9, and matplotlib 3.10.6.

#### 4.4.1. Preprocessing and ROI Extraction

Regions of interest (ROIs) were manually delineated from the acquired hyperspectral images using a custom-developed visualization application. To facilitate this process, hyperspectral data were displayed as pseudo-RGB composites using bands 70 (602 nm), 52 (549 nm), and 19 (452 nm) for the red, green, and blue channels, respectively, with percentile-based normalization (2nd–98th percentile). Users interactively selected ROIs as either rectangular bounding boxes or free-form polygons to accurately encompass the turfgrass samples. The corresponding spectral data were extracted and saved as NumPy (.npy) arrays alongside coordinate metadata for subsequent processing.

#### 4.4.2. Unsupervised Learning and Classification

Following the established protocol, the extracted spectral data were reshaped into a two-dimensional matrix and normalized using a MinMaxScaler from scikit-learn. To reduce the high dimensionality of the spectral data while preserving intrinsic data patterns, Uniform Manifold Approximation and Projection (UMAP) was applied. UMAP parameter settings were selected to preserve local spectral neighborhood structure while yielding visually stable two-dimensional separation of vegetation and non-vegetation clusters.

Subsequently, Density-Based Spatial Clustering of Applications with Noise (DBSCAN) was applied to the UMAP-reduced coordinates. Epsilon (ε) values were adjusted independently for each image region and selected as the lowest value that maintained continuous vegetation clustering without excessive fragmentation or cluster merging. Points identified as noise were remapped to the background class. The cluster labels generated by DBSCAN served as training labels for a Random Forest Classifier (RFC). RFC settings were then chosen to maximize agreement with the DBSCAN-derived vegetation class while minimizing obvious inclusion of soil and shadow pixels under visual inspection.

#### 4.4.3. Refinement via Pseudo-RGB Intersection Masking

To further refine the classification accuracy and eliminate residual non-plant artifacts, a post-processing masking procedure was implemented by computing the intersection of the RFC output and color-based thresholding derived from the pseudo-RGB imagery. Target RGB triplets representing healthy vegetation were sampled directly from the pseudo-RGB composite. A color similarity mask was then generated by including pixels that fell within a defined tolerance range (typically ±30% per channel) of these target RGB values. The final vegetation mask was defined as the spatial intersection of two criteria: (1) pixels classified as the target class by the RFC model, and (2) pixels satisfying the pseudo-RGB color similarity constraints. This combined mask was applied to the original hyperspectral data cube, ensuring that only spectrally and visually validated vegetation pixels were retained for analysis, while masked-out regions were set to zero.

#### 4.4.4. Vegetation Index-Based Filtering and Calculation

Vegetation indices were computed from the filtered .npy data to characterize the spectral properties of the selected regions. A total of eleven vegetation indices were calculated, and their specific spectral band formulations are detailed in Table 3. Each index was normalized to its respective range, and user-defined thresholds were applied to generate binary masks. The final vegetation-filtered regions were defined by the intersection of all enabled vegetation index masks. The mean reflectance for each ROI was then determined by averaging the spectral data of valid pixels within these refined regions.

#### 4.4.5. Independent Point-Based Validation of the Final Vegetation Classification

To independently evaluate the final vegetation classification module, 50 pseudo-RGB images were randomly selected from the representative validation set derived from the selected genotypes. For each image, 200 reference points were randomly sampled and manually annotated as vegetation or background using a custom visualization interface while inspecting the pseudo-RGB image together with the corresponding axis and vegetation index mask layers. In total, 10,000 reference points were annotated and compared with the automated classification output. Performance was quantified using overall accuracy, precision, recall, specificity, F1-score, and Cohen’s kappa. To assess the effect of canopy density on classification performance, images were additionally grouped into high-, intermediate-, and low-coverage subsets.

### 4.5. Data Processing and Representative Genotype Selection

Prior to detailed comparative analysis, a data quality control (QC) procedure was conducted on the initial dataset of 464 genotypes. Genotypes with insufficient replicates (*n* < 3) due to image acquisition errors were excluded to ensure statistical reliability. Consequently, a total of 405 valid genotypes were utilized for the ranking process.

To select representative genotypes for validation, a Combined Ranking Score (CRS) was calculated. The CRS combined physiological quality (represented by five vegetation indices: PSSRb, NDVI, GCI, RE-NDVI, and VOG-REI1) and growth quantity (represented by vegetation pixel count derived from VI frequency). The final rank was determined by applying a weight of 5.0 to the Area rank to balance the contribution of the five quality indices (50:50 ratio), as shown in the following equation:

$$CRS=\;\frac{\sum {Rank}_{Indices}+({Rank}_{Area}\;\times \;5.0)}{10}$$

**Figure 8:**
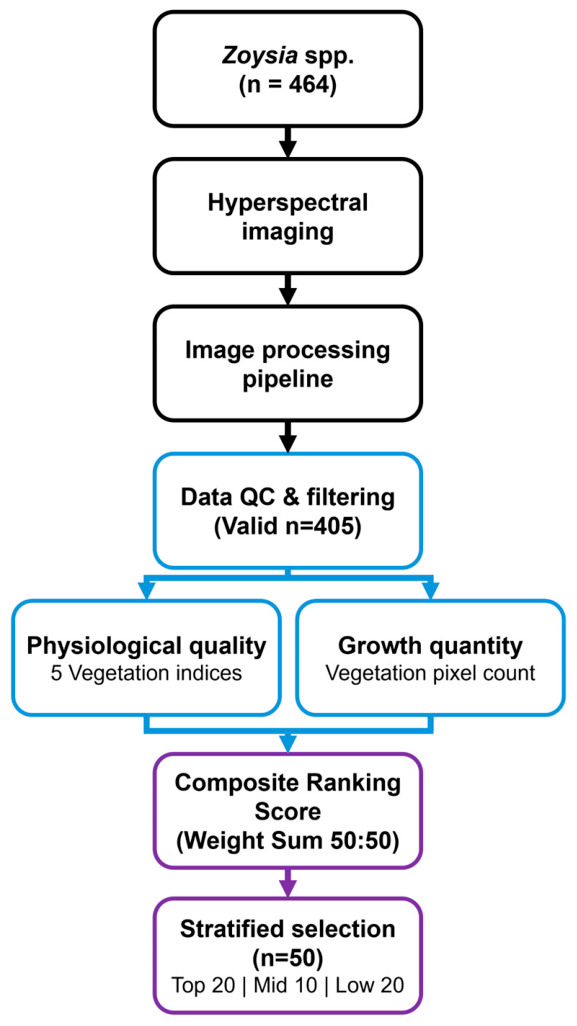
Flowchart of the experimental workflow for high-throughput phenotyping and selection. Black, blue, and purple boxes indicate data acquisition, trait extraction, and selection stages, respectively. A Combined Ranking Score (CRS) integrating physiological quality (vegetation indices) and growth quantity (vegetation pixel count) with equal weighting (50:50) was used to select 50 representative genotypes.

Based on the CRS, 50 representative genotypes were selected to represent the range of phenotypic variation: the Top 20 (Superior), Middle 10 (Intermediate), and Bottom 20 (Inferior) (Figure 8).

### 4.6. Frequency Distribution and Growth Stage Comparison

To quantify temporal changes in vegetation index distributions, frequency histograms were generated for each index. Frequency values, expressed as absolute pixel counts, were binned according to the specific index ranges listed in Table 3. For the selected 50 lines (*n* = 3), mean frequency distributions were obtained by averaging the frequency arrays for each variety and time period. The histogram area difference (∆A) between the two measurement periods was calculated as follows:

$$∆A=\;\sum_{i=1}^{n}\left(f_{Oct,i}-f_{Jul,i}\right)$$

where $f_{Oct,i}$ and $f_{Jul,i}$ represent the pixel frequency at bin i for the second (October, 16 WAT) and first (July, 6 WAT) measurement periods, respectively. Positive values indicate an overall increase in pixel counts associated with the vegetation index (canopy expansion), while negative values indicate a decrease.

## 5. Conclusions

This study evaluated hyperspectral imaging as a high-throughput phenotyping tool for seasonal growth assessment in a large Zoysia breeding population. A user-in-the-loop hybrid segmentation pipeline integrating UMAP, DBSCAN, Random Forest classification, and pseudo-RGB refinement enabled effective vegetation extraction from field-nursery images. Independent validation against 10,000 manually annotated reference points showed strong overall agreement between the automated module and manual reference labels, although performance decreased under high-coverage conditions, indicating increased mixed-pixel ambiguity in dense canopies. CRS was significantly associated with aerial shoot count and runner count, suggesting that the framework can support the relative ranking of density-related vegetative growth traits. Because the present results were obtained from a single location and one growing season, and the correlations with manual traits were moderate, the proposed framework is best interpreted as a screening and ranking tool rather than a direct predictive model. Additional validation across environments, canopy structures, and Zoysia species will be needed to assess broader applicability.

## Figures and Tables

**Table 1:** Independent point-based validation of the final vegetation classification module against manually annotated reference points.

Coverage Group	N Points	Overall Accuracy	Precision	Recall	Specificity	F1-Score	Cohen’s Kappa
Overall	10,000	0.9697	0.8830	0.9240	0.9779	0.9030	0.8851
High coverage	4800	0.9565	0.8477	0.9186	0.9646	0.8817	0.8551
Intermediate coverage	2400	0.9746	0.9150	0.9124	0.9853	0.9137	0.8988
Low coverage	2800	0.9882	0.9479	0.9508	0.9931	0.9493	0.9426

**Table 2:** Morphological characteristics of the Top 20, Bottom 20, and total population (*n* = 405) based on the Combined Ranking Score (CRS). Values are presented as mean ± SD. Significance levels: *** *p* < 0.001; ns, not significant.

Group	Top 20	Bottom 20	Total (*n* = 405)	Sig
Aerial shoot count (ea)	84.1 ± 27.8	25.9 ± 6.7	46.9 ± 22.3	***
Runner count (ea)	2.4 ± 1.4	0.7 ± 0.7	1.2 ± 1.7	***
Plant height (cm)	5.1 ± 4.4	5.6 ± 1.3	5.2 ± 1.6	ns

**Table 3:** Vegetation indices calculated from hyperspectral imagery.

Index	Formula	References
NDVI	$\frac{NIR-Red}{NIR+Red}$	[45]
EVI	$2.5\times \frac{NIR-Red}{NIR+6\times Red-7.5\times Blue+1}$	[46]
GNDVI	$\frac{NIR-Green}{NIR+Green}$	[47]
RE-NDVI	$\frac{NIR-RE}{NIR+RE}$	[48]
mND705	$\frac{R_{750}-R_{705}}{R_{750}+R_{705}-2\times R_{445}}$	[49]
GCI	$\frac{NIR}{Green}-1$	[50]
VOG-REI 1	$\frac{R_{740}}{R_{720}}$	[51]
SRI	$\frac{NIR}{Red}$	[52]
PSSRa	$\frac{R_{800}}{R_{675}}$	[53]
PSSRb	$\frac{R_{800}}{R_{650}}$	[53]
PSSRc	$\frac{R_{800}}{R_{500}}$	[53]

Abbreviations: NDVI, Normalized Difference Vegetation Index; EVI, Enhanced Vegetation Index; GNDVI, Green Normalized Difference Vegetation Index; RE-NDVI, Red-Edge Normalized Difference Vegetation Index; mND705, modified Normalized Difference index at 705nm; GCI, Green Chlorophyll Index; VOG-REI 1, Vogelmann Red Edge Index 1; SRI, Simple Ratio Index; PSSRa/b/c, Pigment-Specific Simple Ratio (a/b/c); NIR, near-infrared reflectance; RE, red-edge reflectance; *R_xxx_*, reflectance at wavelength x nm.

## Data Availability

The data presented in this study are available upon request from the corresponding author.
